# Molecular insights into the premature aging disease progeria

**DOI:** 10.1007/s00418-016-1411-1

**Published:** 2016-02-04

**Authors:** Sandra Vidak, Roland Foisner

**Affiliations:** Max F. Perutz Laboratories (MFPL), Department of Medical Biochemistry, Vienna Biocenter (VBC), Medical University Vienna, Dr. Bohr-Gasse 9/3, 1030 Vienna, Austria

**Keywords:** Lamins, Premature aging, Progeria, Nucleoplasmic lamins, Chromatin, Signaling, Adult stem cells, Senescence

## Abstract

Hutchinson–Gilford progeria syndrome (HGPS) is an extremely rare premature aging disease presenting many features resembling the normal aging process. HGPS patients die before the age of 20 years due to cardiovascular problems and heart failure. HGPS is linked to mutations in the *LMNA* gene encoding the intermediate filament protein lamin A. Lamin A is a major component of the nuclear lamina, a scaffold structure at the nuclear envelope that defines mechanochemical properties of the nucleus and is involved in chromatin organization and epigenetic regulation. Lamin A is also present in the nuclear interior where it fulfills lamina-independent functions in cell signaling and gene regulation. The most common *LMNA* mutation linked to HGPS leads to mis-splicing of the *LMNA* mRNA and produces a mutant lamin A protein called progerin that tightly associates with the inner nuclear membrane and affects the dynamic properties of lamins. Progerin expression impairs many important cellular processes providing insight into potential disease mechanisms. These include changes in mechanosignaling, altered chromatin organization and impaired genome stability, and changes in signaling pathways, leading to impaired regulation of adult stem cells, defective extracellular matrix production and premature cell senescence. In this review, we discuss these pathways and their potential contribution to the disease pathologies as well as therapeutic approaches used in preclinical and clinical tests.

## Introduction

Aging is a universal process in biological organisms that is characterized by a time-dependent progressive decline in cellular and tissue function. At the molecular and cellular level, nine hallmarks have been proposed to contribute to the extremely complex, multifactorial process of aging: genomic instability and defects in nuclear architecture, telomere attrition, epigenetic alterations and chromatin remodeling, loss of proteostasis, deregulated nutrient sensing, mitochondrial dysfunction, cellular senescence, stem cell exhaustion and altered intercellular communication (Lopez-Otin et al. 2013). Aging represents a major risk factor for the development of several diseases including cancer and cardiovascular and neurodegenerative diseases (Campisi et al. 2011; Niccoli and Partridge 2012).

Several premature aging-like syndromes have been described in humans, presenting many features that resemble normal aging (Navarro et al. 2006). Progeroid syndromes represent a group of rare genetic disorders with features of premature aging (Ghosh and Zhou 2014; Pereira et al. 2008). They are “segmental disorders” that affect multiple organs and tissues and display some but not all symptoms observed in physiological aging (Ghosh and Zhou 2014; Sahin and Depinho 2010; Navarro et al. 2006). Most of these syndromes have been well characterized, and a number of associated genes and causative mutations have been identified in recent years (Kudlow et al. 2007; Pereira et al. 2008). Several heritable premature aging syndromes have been linked to mutations in genes encoding DNA repair proteins such as Werner syndrome (WS), Cockayne syndrome (CS), Bloom syndrome (BS), ataxia-telangiectasia (A-T), xeroderma pigmentosum (XP) and Rothmund–Thomson syndrome (RTS), suggesting that the maintenance of genome integrity has a central role in human aging (Navarro et al. 2006; Pereira et al. 2008). In addition, mutations in the mitochondrial DNA (mtDNA) and impairment of mitochondrial pathways were shown to lead to the development of progeroid phenotypes in the mtDNA mutator premature aging mouse model (Trifunovic et al. 2004; Bratic and Larsson 2013).

A different group of genetic premature aging disorders is linked to mutations in the genes encoding A-type lamins or lamin-processing enzymes, including Hutchinson–Gilford progeria syndrome (HGPS) and restrictive dermopathy (RD) (Navarro et al. 2005; De Sandre-Giovannoli et al. 2003). HGPS has attracted much attention not only because of the severity of the disease, but also due to the hypothesis that the expression of the disease-causing lamin A variant called progerin may also be linked to the normal aging process. While in the most common form of HGPS a silent point mutation in the *LMNA* gene affects splicing of prelamin A mRNA and leads to the production of the disease-causing lamin A variant progerin (De Sandre-Giovannoli et al. 2003; Eriksson et al. 2003), the sporadic use of the same cryptic splice site in wild-type *LMNA* can lead to the production of mis-spliced prelamin A mRNA and progerin also in cells and tissues of aged healthy individuals (Scaffidi and Misteli 2006; McClintock et al. 2007). Furthermore, HGPS and normal aging share many cellular phenotypes, such as abnormal nuclear shape, loss of epigenetic marks and increased DNA damage, as well as tissue pathologies including reduced bone density and cardiovascular disease (Burtner and Kennedy 2010). Thus, better understanding of the molecular pathogenesis underlying progeroid syndromes can lead to a better understanding of the normal human aging process. In this review, we summarize the genetic cause of HGPS and consequences for posttranslational lamin processing and lamin functions. We also describe potential causative disease mechanisms and how they may contribute to the cellular, tissue and organismal phenotypes. Finally, we briefly summarize potential strategies for treatment of HGPS.

## Nuclear lamins: biochemistry, functions and link to disease

Lamins are type V intermediate filament proteins expressed in all metazoan cells. They are the major building blocks of the nuclear lamina, a complex filamentous meshwork underneath the inner nuclear membrane (INM) (Dechat et al. 2010a; Gruenbaum and Foisner 2015). Lamins share with their cytoskeletal counterparts the domain organization, encompassing a ~45-nm-long central α-helical rod domain flanked by two globular domains (Coulombe et al. 2001; Herrmann et al. 2007), but they contain additional lamin-specific motifs and domains in the C-terminus, such as a nuclear localization signal, a highly conserved immunoglobulin (Ig)-like fold and in most cases a CaaX-box (C = cysteine, a = aliphatic residue, X = any amino acid) (Dechat et al. 2010a; Gruenbaum and Foisner 2015). Based on their sequence similarities, biochemical and structural properties and their expression patterns during development, lamins are classified into A- and B-types. B-type lamins are expressed throughout development, whereas A-type lamins are weakly or not at all expressed in early embryonic stages and in embryonic stem cells (Eckersley-Maslin et al. 2013), but they are upregulated at later stages during development (Gruenbaum and Foisner 2015). In mammals, *LMNB1* and *LMNB2* encode the two major B-type lamins, lamin B1 and B2, respectively, and *LMNB2* encodes an additional smaller germ cell-specific isoform (lamin B3). A-type lamins are derived from a single *LMNA* gene by alternative splicing, which gives rise to the two major A-type isoforms (lamin A and the smaller splice variant lamin C) and two less abundant isoforms, the germ cell-specific lamin C2 and lamin AΔ10 (Broers et al. 2006; Dechat et al. 2010a). Lamin B1 and B2 and lamin A are expressed as prelamins and undergo several steps of posttranslational processing at their C-terminal–CaaX sequence (Young et al. 2005). The first three processing steps are common to B-type lamins and lamin A and include the addition of a farnesyl group to the C-terminal cysteine residue by farnesyltransferase (FTase) followed by cleavage of the -aaX tripeptide by *FACE1*/ZMPSTE24 or *FACE2*/Rce1 proteases, and carboxymethylation of the farnesylated cysteine residue by the isoprenyl-cysteine-carboxy-methylatransferase (ICMT) (Rusinol and Sinensky 2006). The processing of B-type lamins stops at this step, resulting in mature lamin B with a C-terminal farnesyl- and carboxymethyl group. The hydrophobic farnesyl group mediates strong interaction with the INM, leading to the predominant localization of B-type lamins at the nuclear periphery. In contrast, farnesylated prelamin A is further processed by *FACE1*/ZMPSTE24, which removes the 15 C-terminal amino acids including the farnesylated and carboxymethylated cysteine residue (Pendas et al. 2002). As a consequence, mature lamin A as well as lamin C, which lacks a CaaX-box and never becomes farnesylated, lack the hydrophobic farnesyl group and are therefore not only found at the peripheral lamina associated with the INM, but they can also localize to the nuclear interior (Dechat et al. 2010b; Kolb et al. 2011; Moir et al. 2000; Naetar et al. 2008). In proliferating cells, the nucleoplasmic pool of lamin A/C accounts for 10–15 % of total lamin A/C and is highly mobile compared to lamin A/C at the periphery (Moir et al. 2000).

Lamins have long been known as structural components providing mechanical support for the nucleus (Gruenbaum and Foisner 2015), and recent reports showed that lamins define the mechanochemical properties of the nucleus (Osmanagic-Myers et al. 2015); lamin A is responsible for nuclear stiffness, and B-type lamins for nuclear elasticity (Buxboim et al. 2014; Swift et al. 2013). Besides their mechanochemical role, lamins have a multitude of additional functions, including chromatin organization, gene regulation, DNA repair, and (mechano-) signaling (Amendola and van Steensel 2014; Andres and Gonzalez 2009; Dechat et al. 2010a; Dittmer and Misteli 2011; Gruenbaum and Foisner 2015; Ho and Lammerding 2012).

The multitude of functions of nuclear lamins can, at least in part, be explained by their interactions with a plethora of lamin-binding proteins at the nuclear envelope (Brachner and Foisner 2011; Gruenbaum and Foisner 2015; Wilson and Foisner 2010). It is generally assumed that ubiquitously expressed lamins together with their differentially expressed binding partners form “functional units” at the nuclear envelope responsible for diverse, tissue-specific roles of lamins (Korfali et al. 2012; Worman and Schirmer 2015). In contrast to the huge number of lamin-binding proteins at the nuclear envelope, only a few proteins are known to interact with the mobile nucleoplasmic pool of lamin A/C to form “functional units” in the nuclear interior. The best-characterized interaction partner of A-type lamins in the nuclear interior is lamina-associated polypeptide (LAP) 2α (Dechat et al. 2000; Markiewicz et al. 2002; Dorner et al. 2006; Naetar et al. 2008), a unique isoform of the LAP2 family. Unlike the other LAP2 isoforms, which are integral membrane proteins of the INM, LAP2α lacks a transmembrane domain and localizes in the nuclear interior, where it interacts with and stabilizes nucleoplasmic lamin A/C (Dechat et al. 2000; Naetar et al. 2008). Nucleoplasmic lamin A/C–LAP2α complexes have been implicated in the retinoblastoma protein-mediated regulation of cell proliferation and differentiation of tissue progenitor cells (Dorner et al. 2006; Markiewicz et al. 2002; Naetar et al. 2008) and in chromatin organization (Bronshtein et al. 2015; Zhang et al. 2013).

Given the multitude of functions of the lamins, it is not surprising that mutations in lamins and lamin-binding proteins are associated with a variety of human diseases exhibiting complex patterns of tissue-specific pathologies (Broers et al. 2006; Worman 2012). The majority of diseases are caused by mutations in the *LMNA* gene and are collectively termed laminopathies. Until today more than 500 mutations have been described in *LMNA* (www.umd.be/LMNA/) that give rise to four major groups of diseases with overlapping pathologies, including striated muscle diseases, lipodystrophic syndromes, peripheral neuropathy and accelerated aging disorders (Worman 2012).

## Hutchinson–Gilford progeria syndrome: genetics and cellular and clinical phenotypes

Hutchinson–Gilford progeria syndrome (HGPS) is an extremely rare sporadic autosomal-dominant genetic disorder affecting 1 in 4–8 million newborns and displays phenotypic features of premature aging (Ghosh and Zhou 2014; Gordon et al. 2014). Children with HGPS appear normal at birth but start to exhibit many distinctive clinical features within the first year of life. Classical progeria symptoms include severe growth retardation, loss of hair and subcutaneous fat, prominent eyes and scalp veins, aged-looking skin, joint stiffness and reduced bone density. As children get older they suffer from osteoporosis, atherosclerosis and cardiovascular diseases as the most severe aspect of the disease. HGPS patients die at an average age of 14 years due to myocardial infarction, heart failure or progressive atherosclerosis (Cau et al. 2014; Kieran et al. 2007; Muchir and Worman 2010).

Classical HGPS is caused by a de novo heterozygous mutation (1824C>T, p.G608G) in exon 11 of *LMNA* (De Sandre-Giovannoli et al. 2003; Eriksson et al. 2003), which activates a cryptic splice donor site, resulting in the production of a prelamin A mRNA that contains an internal deletion of 150 base pairs. This transcript is translated into a mutant lamin A protein termed progerin, which harbors a deletion of 50 amino acids within its C-terminus including the *FACE1*/ZMSPTE24 cleavage site (Eriksson et al. 2003). As a consequence, progerin cannot undergo the final proteolytic processing step and permanently retains the C-terminal farnesyl group, leading to its stable association with the INM and predominant localization at the nuclear periphery (Dechat et al. 2007; Davies et al. 2009).

**Fig. 1:**
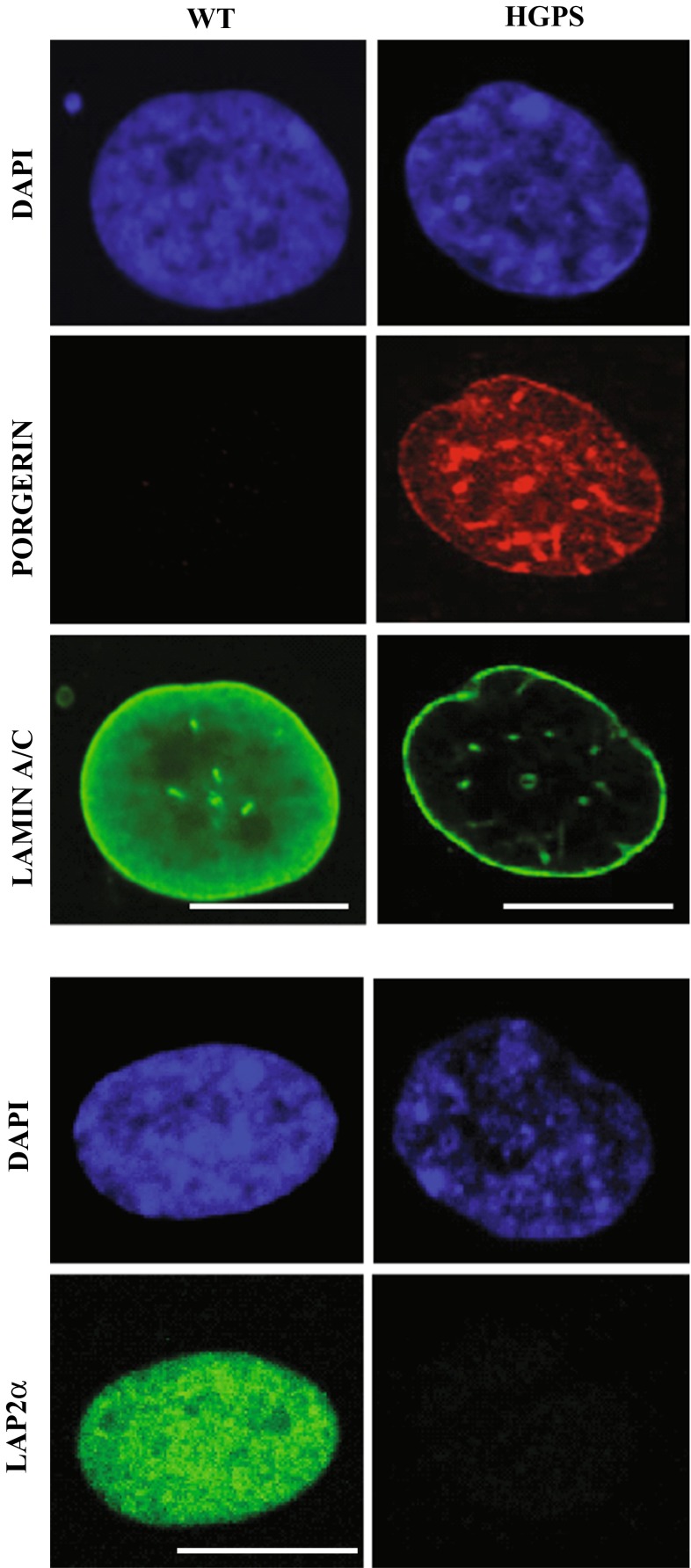
Progerin expression causes loss of nucleoplasmic lamin A/C and LAP2α in primary HGPS fibroblasts. Immunofluorescence analysis of wild-type (WT) and HGPS primary human fibroblasts using anti-progerin (*red*), anti-lamin A/C (*green*) and anti-LAP2α (*green*) antibodies shows significant decrease in the nucleoplasmic pool of A-type lamins and LAP2α levels upon progerin expression. *Scale bar* 10 µm

Progerin is expressed in multiple tissues, mostly of mesenchymal origin including skin, bone, skeletal muscle, adipose tissue, heart and large and small arteries (Gordon et al. 2014). Expression of progerin induces various cellular defects in a dominant-negative manner, including highly lobulated nuclei with thickened lamina, loss of peripheral heterochromatin, accumulation of DNA damage, telomere aberrations and mitochondrial dysfunction, leading to differentiation defects and premature cellular senescence (Bridger and Kill 2004; Brunauer and Kennedy 2015; Goldman et al. 2004; Gonzalo and Kreienkamp 2015; McCord et al. 2013; Scaffidi and Misteli 2008; Shumaker et al. 2006; Vidak et al. 2015). In addition, progerin expression leads to decreased expression levels of lamin B1, heterochromatin protein 1 α (HP1α) and LAP2α, and loss of nucleoplasmic lamins (Fig. 1) (Cenni et al. 2011; Scaffidi and Misteli 2005; Miller et al. 2013; Scaffidi and Misteli 2008; Vidak et al. 2015). These changes together with the abnormal nuclear morphology are often used as cellular disease markers to test therapeutic strategies in cell and mouse models (Cao et al. 2011b; Capell et al. 2005; Fong et al. 2006; Scaffidi and Misteli 2005).

In addition to the classical 1824C>T HGPS mutation, other heterozygous, homozygous or compound heterozygous mutations in *LMNA* have been reported, such as T10I, A57P, L59R, R133L, L140R, S143F, E145K, V169fsX176, D300N, E578V and R644C and c.412G>A (Kirschner et al. 2005; Csoka et al. 2004; Chen et al. 2003; Caux et al. 2003; Jacob et al. 2005; Mory et al. 2008; McPherson et al. 2009; Doh et al. 2009; Renard et al. 2009; Doubaj et al. 2012; Kane et al. 2013), all causing atypical progeroid syndromes (APS). APS mutations affect similar tissues (bone, skin, hair and body fat) and cause similar pathologies (growth retardation, alopecia, tight skin and beaked nose) as classical HGPS, but the course and severity of the symptoms vary greatly (Garg et al. 2009; Doubaj et al. 2012). Compound heterozygous mutations in *FACE,* leading to the complete loss of function of ZMPSTE24 protease and accumulation of farnesylated prelamin A causes autosomal recessive restrictive dermopathy (RD), a progeroid syndrome associated with neonatal death (Navarro et al. 2005). In 2011, a new autosomal recessive syndrome was identified and named Nestor–Guillermo progeria syndrome (NGPS) (Cabanillas et al. 2011). Patients with NGPS display several pathologies similar to HGPS but with a slow clinical course and relatively long survival. NGPS is caused by a homozygous missense mutation in *BANF1* that encodes barrier-to-autointegration factor (BAF), a chromatin protein, which directly interacts with lamins and lamin-binding proteins and has been implicated in chromatin organization (Margalit et al. 2007).

## Mouse models of HGPS

In the search for molecular disease mechanisms of HGPS, several progeroid mouse models have been created. One of the first of these mouse models was generated by knocking-in a mutant *Lmna* allele (*Lmna*^*HG*^) that produces exclusively progerin but no wild-type lamin A and lamin C (Yang et al. 2005). This knock-in mouse model displays phenotypes similar to HGPS children including loss of hair (alopecia) and subcutaneous fat, osteoporosis and premature death, but no cardiovascular defects were reported. In contrast, a transgenic mouse model that carries the mutated G608G human *LMNA* allele on a bacterial artificial chromosome (G608G BAC) develops progressive loss of vascular smooth muscle cells (VSMCs), a feature described also in HGPS patients, but did not show most of the other pathologies (Varga et al. 2006). Another mouse model, in which a point mutation in *Lmna* caused loss of exon 9 (*Lmna*^*L530P/L530P*^, also known as *Lmna*∆*9*), also displayed phenotypes overlapping with HGPS (Mounkes et al. 2003; Hernandez et al. 2010), but the mechanism is still unclear and may differ from that of the classical HGPS.

These mouse models phenocopy some of the phenotypes observed in the HGPS patients, but they do not recreate the exact molecular changes occurring at the *LMNA* locus in HGPS patients. Therefore, Osorio et al. created a mouse knock-in strain that carries a HGPS mutation in the mouse *Lmna* gene (*Lmna*^*G609G*^; 1827C>T; Gly609Gly) and produces progerin due to abnormal splicing of the endogenous *Lmna* mRNA like in HGPS patients (Osorio et al. 2011). These mice phenocopy the main clinical manifestations of human HGPS and open new avenues towards investigating the splicing defect in HGPS and identification of drugs that may correct faulty splicing of prelamin A mRNA in HGPS.

In addition to these transgenic mice expressing progerin ubiquitously, several mouse models with tissue-specific progerin expression have been generated. A transgenic mouse model that contains a human G608G *LMNA* minigene mutant under the control of a tet-operon (tetopLA^G608G^) allowed tissue-specific and inducible expression of progerin by crossing them with transgenic mice expressing the transactivator in specific tissues (Sagelius et al. 2008). Expression of progerin in keratin 5-expressing cells in the epidermis caused loss of subcutaneous fat, fibrosis of the dermis, incomplete development of sebaceous glands, dental problems, hair thinning, decreased stem cell population in epidermal tissues and impaired wound healing ability, supporting the hypothesis that an impaired regeneration capacity of epidermal stem cells may contribute to the HGPS phenotype (Rosengardten et al. 2011). The osteoblast-specific expression of progerin reduced bone density and caused spontaneous fractures most likely due to an abnormal osteoblast differentiation (Schmidt et al. 2012). In contrast, expression of progerin in the aged brain, which usually expresses low levels of lamin A/C, caused some structural nuclear abnormalities but no signs of impaired brain function were detected (Baek et al. 2015).

Deletion of *Zmpste24,* the metalloproteinase involved in the posttranslational maturation of prelamin A, results in the expression of farnesylated prelamin A and produces various progeroid phenotypes in mice. *Zmpste24*^−/−^ mice are normal at birth, but within 4–6 weeks of age they start to display many of the progeroid phenotypes such as growth retardation, alopecia, loss of adipose tissue, multiple spontaneous bone fractures, abnormal nuclear morphology and premature death, as well as muscular dystrophy and dilated cardiomyopathy (Bergo et al. 2002; Pendas et al. 2002). The latter phenotypes may also be linked to the fact that no mature lamin A is produced in these mice, while HGPS phenotypes may be caused by the accumulation of farnesylated prelamin A. In support of this hypothesis, blocking prelamin A farnesylation in these mice by farnesyltransferase inhibitors (FTIs) improves some of the HGPS phenotypes (Fong et al. 2006). Furthermore, a knock-in mouse model that exclusively expresses non-farnesylated prelamin A (*Lmna*^*nPLAO/nPLAO*^) shows no progeroid phenotypes but develops severe cardiomyopathy (Davies et al. 2010). In contrast, observations in *Lmna*^*HG/*+^ mice that FTI treatment ameliorates but not completely abolishes progeria phenotypes suggested that the non-farnesylated form of progerin may retain some toxic features relevant for HGPS. To test this hypothesis, a mouse model exclusively expressing the non-farnesylated form of progerin (*Lmna*^*nHG*^) was generated by replacing the cysteine in the CaaX motif in the *Lmna*^*HG*^ allele with a serine (CSIM → SSIM). Interestingly, homozygote and heterozygote *Lmna*^*nHG*^ mice showed similar, but less severe progeroid phenotypes than those described for *Lmna*^*HG*^ mice (Yang et al. 2008). To exclude that the cysteine-to-serine substitution may itself be toxic, an alternative non-farnesylated progerin-expressing mouse model was generated by deleting the isoleucine in the CSIM motif to create a protein ending in CSM rather than SSIM (*Lmna*^*csmHG*^). Surprisingly, *Lmna*^*csmHG*^ mice showed no noticeable progeroid defects, suggesting that cysteine-to-serine substitution in the *Lmna*^*nHG*^ mouse model may be toxic, although the authors do not exclude the possibility that toxicity of progerin is linked to the 50 amino acid deletion in its C-terminus independent of farnesylation and the deletion of the isoleucine in CSIM may neutralize the toxicity of the protein (Yang et al. 2011b).

## Molecular and cellular mechanisms contributing to the HGPS pathologies

**Fig. 2:**
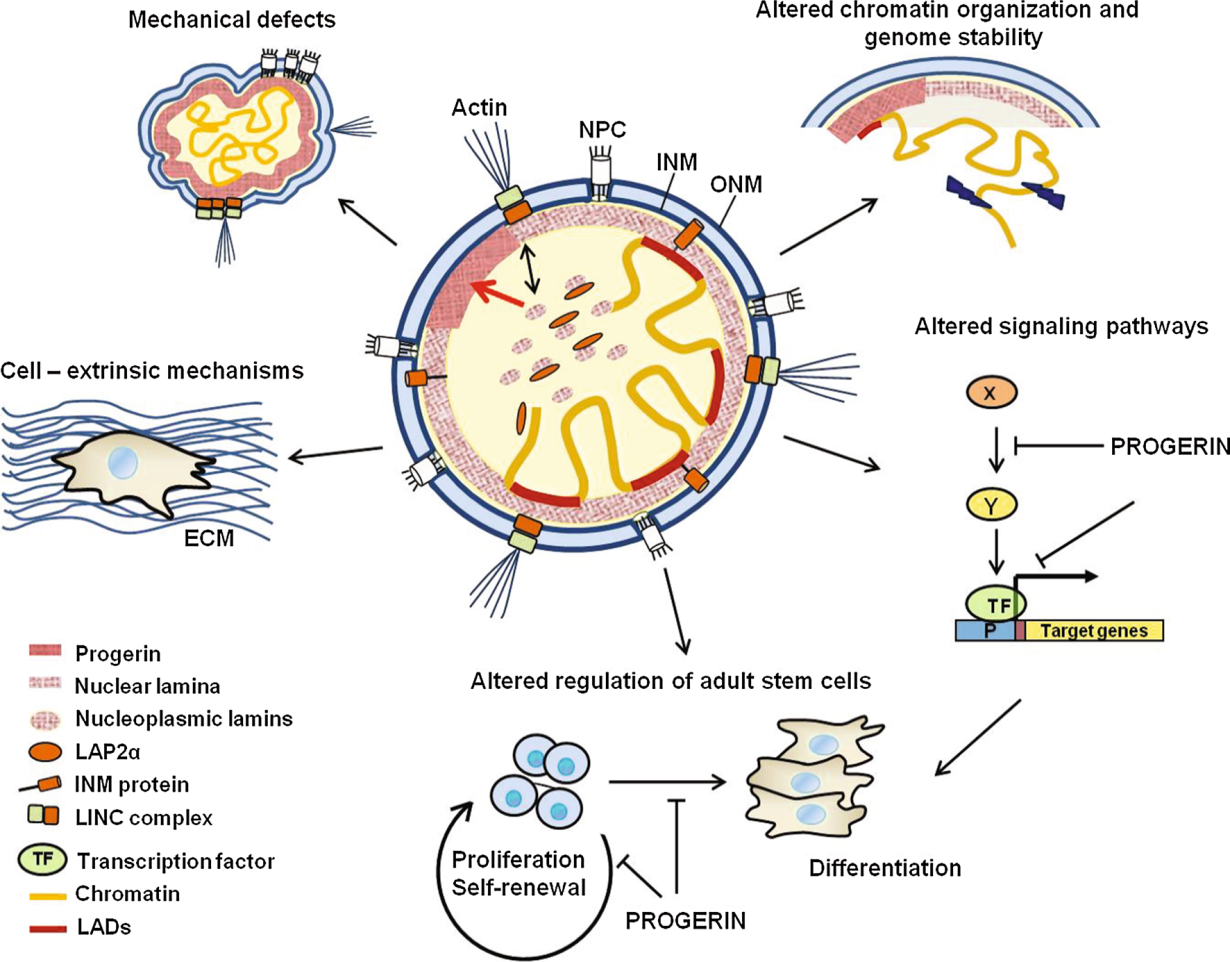
Cellular functions affected by the expression of the lamin A mutant protein progerin. Progerin accumulates at the nuclear membrane/lamina leading to reduced levels of nucleoplasmic lamin A/C–LAP2α. These changes in lamin dynamics affect mechanical properties and mechanosignaling, lead to dissociation of heterochromatic lamina-associated domains (LADs) in the genome from the lamina, and impair signaling pathways and gene expression, all contributing to defects in self-renewal and differentiation of adult stem cells, the production of a faulty extracellular matrix (ECM) and cell senescence

HGPS-causing mutations have been shown to affect many fundamental cellular processes, but how these contribute to the described pathologies is not completely understood. In most cases, we do not even know the detailed molecular mechanisms how progerin expression affects the molecular processes found to be impaired in HGPS. However, based on what is known about lamin A/C and progerin biochemistry, function and interactions (Kubben et al. 2010a, b), one may hypothesize about potential pathways and mechanisms (Fig. 2) discussed below.

### Mechanical defects in HGPS

The presence of the farnesyl group in progerin is thought to be a predominant toxic feature in the pathogenesis of the disease. In healthy cells, A- and B-type lamins form distinct homopolymers and micro-domains at the nuclear periphery (Shimi et al. 2008), but this segregation may be lost in HGPS cells (Delbarre et al. 2006) due to the stable association of permanently farnesylated progerin with the membrane (Dechat et al. 2007). Progressive progerin accumulation at the INM during cellular aging of HGPS cells (Eriksson et al. 2003; Fong et al. 2006; Goldman et al. 2004; Varela et al. 2005) leads to immobilization of wild-type lamin A at the lamina, thickening and increased stiffness of the lamina, prominent lobulation of the nuclear envelope, and clustering of nuclear pores (Dahl et al. 2006; Goldman et al. 2004). Overall, these alterations disrupt the structural and functional integrity of the nuclear lamina and may render cells more susceptible to damage through physical stress (Verstraeten et al. 2007; Zhang et al. 2011). While in wild-type cells nuclei respond to shear stress by up-regulation and re-distribution of A-type lamins (Buxboim et al. 2014; Philip and Dahl 2008; Swift et al. 2013), this rearrangement does not work properly in HGPS cells (Dahl et al. 2006). This defect is of particular importance in tissues that are exposed to mechanical stress such as vasculature, bone and joints, which also present some of the most prominent pathologies in HGPS (Gordon et al. 2014; Zhang et al. 2011). In accordance, HGPS skin fibroblasts and VSMCs display increased mechanosensitivity under biomechanical strain (Verstraeten et al. 2008), which may then lead to the observed severe depletion of arterial VSMCs in progeria patients and some progeria mouse models (Stehbens et al. 1999; Varga et al. 2006).

Altered adaptation of progerin-expressing cells to high sheer stress may also be caused by progerin’s negative effect on the expression of many proteins involved in mechanotransduction and cytoskeletal organization, as well as extracellular matrix proteins (ECM) (Song et al. 2014; Brassard et al. 2015). In accordance with this, accumulation of progerin in the ascending aorta of *Lmna*^*G608G*^ knock-in mice causes reduced expression of the cytoskeleatal protein vimentin (Song et al. 2014). As vimentin has a major role in maintaining cellular integrity and affects apoptotic pathways (Song et al. 2014; Moisan and Girard 2006; Barker et al. 2010), the impaired vimentin expression may lead to defects in mechanotransduction and mechanosignaling in progerin-expressing VSMCs (Song et al. 2014; Brassard et al. 2015). Furthermore, progerin expression was shown to impair linker of the nucleoskeleton and cytoskeleton (LINC) complexes at the nuclear envelope through the stabilization and accumulation of SUN1, a LINC component in the INM, and reducing SUN1 mobility (Chen et al. 2014). As the LINC complex is essential for nucleocytoskeletal coupling and mechanotransduction (Osmanagic-Myers et al. 2015), an impaired LINC complex may further contribute to impaired shear stress response in the vasculature of HGS patients.

### Impaired chromatin organization

A-type lamins can directly interact with DNA and histones (Gruenbaum and Foisner 2015), and together with a number of lamin A-binding chromatin proteins, such as members of the LEM domain protein family (Brachner and Foisner 2011; Solovei et al. 2013), they have been implicated in higher-order chromatin organization, heterochromatin formation and epigenetic regulation (Amendola and van Steensel 2014; Dechat et al. 2009; Gonzalez-Suarez and Gonzalo 2010). A-type lamins contribute to the tethering of heterochromatic genomic regions, termed lamina-associated domains (LADs) to the nuclear lamina (Meuleman et al. 2013; Peric-Hupkes et al. 2010) but they also interact with promoter regions of genes, thereby affecting gene expression during cell differentiation (Lund et al. 2013; Ronningen et al. 2015).

It is therefore not surprising that progerin-expressing HGPS nuclei display significant changes in chromatin, such as loss of peripheral heterochromatin, a decrease in the repressive histone marks H3K9me3 and H3K27me3 and an increase in H4K20me3 (Columbaro et al. 2005; Kubben et al. 2012; McCord et al. 2013; Scaffidi and Misteli 2005; Shumaker et al. 2006). It is currently unknown how progerin affects chromatin organization mechanistically, but several reports showing an effect of progerin on epigenetic modifier and chromatin regulator proteins may provide clues towards potential mechanisms. For example, progerin expression causes upregulation of the methyltransferase Suv39h1, while Suv39h1 depletion delays senescence of HGPS cells and prolongs lifespan of *Zmpste24*^−/−^ mice (Liu et al. 2013). Furthermore, progerin-expressing cells have reduced levels of heterochromatin protein 1 (HP1) α (Scaffidi and Misteli 2008) and of several proteins of the nucleosome remodeling NuRD complex (Pegoraro et al. 2009; Meshorer and Gruenbaum 2009; Prokocimer et al. 2013). Interestingly, inactivation of the NuRD complex in wild-type cells can also induce aging-associated chromatin defects resembling those observed in HGPS patients. Changes in chromatin organization and epigenetic regulation in progeria cells may in turn have a profound impact on gene expression and genome stability, thereby contributing to many disease phenotypes (Prokocimer et al. 2013).

Most of the lamin A/C chromatin interaction studies so far have focused on the nuclear lamina, but, given the dual location of A-type lamins at the nuclear lamina and in the nuclear interior, it is tempting to speculate that nucleoplasmic lamins together with their binding partner LAP2α may interact with and regulate chromatin throughout the nucleus (Gesson et al. 2014). In support of this, a recent study demonstrated that only 30 % of all cellular LADs in the genome associate with the nuclear envelope in a given cell, while the rest localizes in the nuclear interior, but LADs are stochastically reshuffled to the nuclear lamina in each cell cycle (Kind et al. 2013). This indicates that lamin A–LAD interaction may also occur in the nuclear interior. In line with this model, lamin A/C-deficient cells show increased mobility of chromatin in the nuclear interior (Bronshtein et al. 2015), indicating that lamin A may cross-link chromatin fibers. Moreover, several recent findings indicate that A-type lamins interact also with genomic regions outside of the LADs (Lund et al. 2013, 2015; Ronningen et al. 2015). Furthermore, LAP2α has been found to interact with chromatin at a genome-wide level and to affect chromatin interaction of high-mobility group N protein 5 (HMGN5), a non-histone protein involved in higher-order chromatin organization (Zhang et al. 2013). As progerin expression reduces levels of nucleoplasmic lamin A/C and LAP2α (Dahl et al. 2006; Scaffidi and Misteli 2005; Vidak et al. 2015), it is conceivable that chromatin organization in the nuclear interior is particularly affected in HGPS. In support of this, re-expression of LAP2α in HGPS cells was found to rescue proliferation of the cells, most likely due to the rescue of the impaired chromatin organization at telomeres (Chojnowski et al. 2015) and at genomic regions harboring ECM genes (Vidak et al. 2015).

### Genome instability

Progerin expression may also affect genome stability by negatively affecting DNA damage repair pathways (Liu et al. 2005). HGPS cells and cells derived from *Zmpste24*^−/−^ mice display impaired recruitment of the DNA double-strand break (DSB) repair factors p53-binding protein 1 (53BP1) and Rad50 and Rad51 to the sites of DNA damage (Liu et al. 2005; Manju et al. 2006), resulting in the accumulation of DSBs (Musich and Zou 2011; Richards et al. 2011). Progerin expression also affects the expression and localization of the nucleotide excision repair protein XPA (Xeroderma Pigmentosum complementation group A) at DNA lesions, resulting in persistent activation of DNA damage response checkpoint kinases ataxia-telangiectasia-mutated (ATM) and ATM and Rad3-related (ATR) protein (Manju et al. 2006; Gonzalo and Kreienkamp 2015). Persistent DNA damage in turn activates tumor suppressor p53 and promotes senescence, one of the phenotypes described as hallmarks of HGPS (Collado et al. 2007).

Deficiencies in DSB repair and telomere dysfunction are major contributors to genome instability in aging cells (Hoeijmakers 2009; Blasco 2005). Dysfunctional telomeres are recognized as DSBs and activate the non-homologous end joining (NHEJ) repair pathway (di Fagagna et al. 2003; Gonzalo and Kreienkamp 2015). Persistent telomere dysfunction and shortening of telomeres below a critical length cause permanent growth arrest known as replicative senescence (Gonzalo and Kreienkamp 2015). HGPS cells were found to undergo accelerated telomere shortening when grown in culture (Decker et al. 2009), and ectopic expression of progerin in wild-type fibroblasts leads to accumulation of DNA damage at telomeres (Benson et al. 2010; Cao et al. 2011a), both resulting in proliferation arrest and senescence. Accordingly, re-expression of telomerase improves proliferation and extends HGPS cellular lifespan (Kudlow et al. 2007; Benson et al. 2010; Chojnowski et al. 2015), suggesting that telomere dysfunction underlies genomic instability and premature senescence in progerin-expressing cells (Gonzalo and Kreienkamp 2015). The molecular mechanisms leading to telomere dysfunction upon progerin expression are poorly understood. Recent studies suggest a role of LAP2α in stabilizing telomere and chromatin structure by increasing the epigenetic H3K27me3 histone mark, preventing progerin-associated DNA damage and rescuing premature senescence (Chojnowski et al. 2015).

### Altered regulation of signaling pathways

Lamins serve as scaffolds for various signaling molecules and transcription factors, thereby regulating their activity (Gruenbaum and Foisner 2015; Osmanagic-Myers et al. 2015). This scaffolding function has a dual role. On the one hand, lamins serve as a platform enabling efficient activation of signaling molecules, such as the extracellular-signal regulated kinase (ERK)-dependent activation of transcription factor c-fos at the lamina (Gonzalez et al. 2008). On the other hand, they sequester various transcription factors and their regulating molecules to the nuclear periphery attenuating their function on target genes (Ivorra et al. 2006; Scaffidi and Misteli 2008). Therefore, it is not surprising that progerin expression causes misregulation of various signaling pathways (Prokocimer et al. 2013) as shown in numerous examples; the molecular mechanisms, however, remain mostly obscure. Fibroblasts from the *Lmna* Δ9 progeria mouse model have reduced Wnt/β-catenin signaling causing defects in the expression of ECM genes (Hernandez et al. 2010). Similarly, hair follicle stem cells in *Zmpste24*^−/−^ mice have reduced levels of active β-catenin (Espada et al. 2008). As wnt signaling is important for cartilage and bone development, an impaired wnt signaling can contribute to the bone phenotype in HGPS patients. Notch signaling, another important pathway for the regulation of cell fate and stem cell differentiation during osteogenesis and adipogenesis (Hori et al. 2013), is also affected in progerin expressing cells (Scaffidi and Misteli 2008). Here, some insight into the potential molecular mechanism of progerin’s effect on Notch-signaling has been provided: Sequestration of the Notch co-activator SKIP (Ski-interacting protein) by wild-type lamins is impaired by progerin, leading to increased activation of major Notch downstream effectors (HES1, HES5, HEY1 and TLE1) (Scaffidi and Misteli 2008). NF-κB, an important transcription factor activated as a response to damage, stress and inflammation (Ghosh and Hayden 2008) and during aging (Tilstra et al. 2012), was found to be hyperactivated in progeroid mice (*Zmpste24*^−*/*−^ and Lmna^G609G^ knock-in mice) (Osorio et al. 2012). Crossing *Zmpste24*^−*/*−^ mice with transgenic mice displaying reduced NF-κB signaling (haploinsufficient for the p65 (RelA) NF-κB subunit, *RelA*^+/−^) or treatment of mice with the NF-κB inhibitor sodium salicylate extended life span and rescued skin and immunological phenotypes (Kawahara et al. 2009; Osorio et al. 2012).

Complexes of nucleoplasmic A-type lamins and LAP2α interact directly with retinoblastoma protein (pRb) a major regulator of cell proliferation (Markiewicz et al. 2002; Dorner et al. 2006), thereby affecting pRb localization and stability (Johnson et al. 2004; Andres and Gonzalez 2009; Nitta et al. 2007) and expression of pRb target genes (Dorner et al. 2006; Naetar et al. 2008). Genome-wide expression studies in primary HGPS-derived dermal fibroblasts revealed an impaired pRb signaling network (Marji et al. 2010). Thus, the loss of nucleoplasmic lamin A/C and LAP2α in HGPS cells (Vidak et al. 2015) may be linked to a deregulation of the pRb pathway in HGPS, leading to impaired regulation of tissue stem cells (Naetar et al. 2008). An impairment of pRb signaling may also be linked to the reduced levels of heterochromatic histone marks in HGPS in view of the described role of pRb in stabilizing histone methylation (Gonzalo et al. 2005).

### Altered regulation of adult stem cells

Adult stem cells constantly replace non-functional and dying cells in many tissues and an age-related decline in their regenerative capacity is an important factor in biological aging (Brassard et al. 2015). Several lines of evidence suggest that A-type lamins may be involved in the regulation of proliferation and differentiation of mesenchymal stem cells (MSCs) as well as tissue progenitor cells (Gotzman and Foisner 2006; Pekovic et al. 2007). MSCs are adult stem cells important for the regeneration of many tissues profoundly affected in HGPS such as bone, skin, muscle and adipose tissue (Scaffidi and Misteli 2008; Dreesen and Stewart 2011; Halaschek-Wiener and Brooks-Wilson 2007). Naive MSCs derived from HGPS patients express low progerin levels in vivo, but accumulate significant amounts of progerin with increasing passages in vitro (Wenzel et al. 2012). Both, adult stem cell self-renewal and differentiation may be affected in HGPS through impaired signaling and chromatin organization as described above. In line with this, *Zmpste 24*^−*/*−^ and tetop-*Lmna*^*G608G;*K5tTA+^ progeria mouse models (see in chapter mouse models) display decreased numbers and altered proliferative capacity of epidermal stem cells (Espada et al. 2008; Rosengardten et al. 2011) and muscle-derived stem/progenitor cells (MDPSCs) (Lavasani et al. 2012). Interestingly, intraperitoneal administration of MDPSCs from young wild-type mice to progeroid mice leads to significant extension of life span and health span (Lavasani et al. 2012), further supporting the hypothesis that an impaired stem cell regulation contributes to premature aging. Postnatal, but not embryonic fibroblasts, derived from the *Lmna∆9* progeria mouse model, show proliferative arrest and premature senescence (Hernandez et al. 2010). Why this phenotype is detectable only in postnatal cells remains unknown, but it is likely linked to an impaired extracellular matrix (ECM) production (Hernandez et al. 2010).

Based on the emerging findings in HGPS, it is tempting to speculate that a misbalance in stem cell self-renewal and differentiation coupled with increased mechanical sensitivity could lead to stem cell exhaustion and inefficient repair of the damaged tissues, contributing to many of the phenotypes in HGPS (Gotzman and Foisner 2006).

### ECM-mediated HGPS mechanisms

Several recent findings led to the newly emerging concept that progerin expression may lead to impaired expression of ECM components and formation of a faulty ECM, which in turn may be causative for many of the cellular phenotypes observed in HGPS. The ECM is well known to have a major role in cell proliferation, differentiation, cell adhesion and migration, and cell survival (Gattazzo et al. 2014; Humphrey et al. 2014), and ECM production is also compromised during physiological aging (Yang et al. 2011a). Abnormal ECM production and rearrangement during development and tissue homeostasis result in many pathological processes including tissue fibrosis and cancer.

Gene expression analyses in HGPS patient fibroblasts showed a profound deregulation of ECM components (Csoka et al. 2004), including decreased levels of ECM remodeling enzymes such as metaloproteinases (MMPs) (Harten et al. 2011; Vidak et al. 2015) and increased levels of type IV and VI collagen and fibronectin (Colige et al. 1991; Maquart et al. 1988; Song et al. 2014). The importance of a faulty ECM in the development of HGPS phenotypes is supported by several observations: Proliferation of human cells expressing progerin (Vidak et al. 2015) and of mouse adult fibroblasts derived from *Lmna*∆*9* progeria mice (Hernandez et al. 2010) is rescued upon growth on ECM derived from wild-type cells. The most convincing data supporting the role of the ECM in disease development in vivo comes from *Zmpste24* mosaic mice containing similar proportion of *Zmpste24*-deficient (prelamin A-accumulating) and *Zmpste24*-proficient (mature lamin A-containing) cells in tissues (de la Rosa et al. 2013). Surprisingly, these mice develop normally and maintain the same proportion of mutant versus wild-type cells in their tissues throughout life, indicating that progeroid *Zmpste24*^−*/*−^ cells develop normally in a background providing normal ECM and possibly other extrinsic factors.

Overall, a deregulation of ECM production and remodeling could account for both an impaired proliferation and differentiation of osteoblasts and chondrocytes during cartilage development (Muchir and Worman 2010), as well as an excessive ECM deposition in the vascular system causing increased arterial stiffness in HGPS (Olive et al. 2010).

## Therapeutic approaches in progeria

Therapeutic approaches for HGPS treatment can be envisaged to work at different levels from genes to tissues, including approaches to correct protein function, RNA splicing and mutations in the *LMNA* gene, to cell replacement strategies and treatments to reverse the cellular phenotypes (Gordon et al. 2014). The first therapy developed for HGPS and already tested in clinical trials (Gordon et al. 2012) aimed at correcting the mutant protein. As the presence of the farnesyl group in progerin was proposed to be the predominant deleterious and toxic component, efforts were put into treatments blocking progerin farnesylation through pharmacologically targeting the isoprenoid biosynthesis pathway (Young et al. 2005; Cau et al. 2014). The initial treatment used drugs called farnesyltransferase inhibitors (FTIs), previously developed as potential anti-cancer drugs, which improve some disease parameters in cultured cells (e.g., nuclear shape and proliferation) and progeria mouse models (Fong et al. 2006; Toth et al. 2005; Yang et al. 2005). Based on these preclinical tests a prospective clinical trial was commenced at Boston Children’s Hospital from 2007 to 2009 including 26 HGPS patients (Gordon et al. 2012). Administration of the FTI drug lonafarnib improved weight gain, bone structure and vascular stiffness, with some of the HGPS patients developing mild drug-related side effects (Gordon et al. 2012). After the initiation of the first clinical trial, it was demonstrated that an alternative prenylation pathway called geranyl geranylation may be activated in the presence of FTIs, offering possible explanations for the only moderate efficiency of the FTI treatment in preclinical trials (Yang et al. 2008). Therefore, the pharmacological strategy was adjusted and tested in the *Zmpste 24*^−*/*−^ progeria mouse model, using a combined aminobisphosphonate (zoledronic acid) and statin (pravastatin) treatment, inhibiting farnesyl-pyrophosphatase synthase and the HMG-CoA reductase, respectively (Varela et al. 2008). Based on promising results in preclinical trials using the *Zmpste 24*^−*/*−^ progeria mouse model (Varela et al. 2008), a second clinical trial combining statins and aminobisphosphonates was initiated from 2008 to 2013 in Marseille’ La Timone Children’s Hospital, but the results of the study are not published yet (Cau et al. 2014).

Another approach for treatment aims at reducing progerin protein levels rather than blocking progerin farnesylation. Two major cellular mechanisms are involved in removing misfolded, mutant or aggregated proteins, proteasomal degradation and autophagy. Although a detailed study on potential pathways involved in progerin degradation has not been done so far, several observations suggest that progerin may be removed by activating macroautophagy. Autophagy is a cytoplasmic degradation machinery that targets damaged proteins and organelles to lysosomal degradation and is upregulated in times of stress such as starvation to provide a source of amino acids (Tanida et al. 2008). Treatment with rapamycin, an inhibitor of the mammalian target of rapamycin (mTOR) pathway, upregulates autophagy and extends life span from yeast to mammals (Johnson et al. 2013; Jung et al. 2010; Madeo et al. 2010). Rapamycin treatment of cultured HGPS cells increases progerin clearance by macroautophagy-related pathways and reduces some of the disease phenotypes, such as lobulated nuclei, LAP2α levels and DNA damage (Cao et al. 2011b; Cenni et al. 2011). Interestingly, it has been suggested that FTIs may indirectly affect mTOR by inhibiting the farnesylation of Rheb GTPase, an upstream activator of mTOR (Hanker et al. 2010).

Sulforaphane, an antioxidant derived from cruciferous vegetables, which stimulates proteasome activity and autophagy in normal and HGPS fibroblast cultures, was also found to enhance progerin clearance by autophagy and to reverse the cellular hallmarks of HGPS (Gabriel et al. 2015). Furthermore, two recent studies suggested that retinoids alone (Kubben et al. 2015) or in a combination with rapamycin (Pellegrini et al. 2015) lower the amount of progerin and rescue progeroid phenotypes in cultured cells. These findings together suggest that compounds acting by decreasing progerin levels in the cell could represent a potent tool for new treatments. Autophagy-activating drugs could be particularly beneficial in progeria treatment, but careful in vivo analyses have to be conducted before including them in clinical trials.

An RNA-targeting therapeutic strategy aims at eliminating or inhibiting alternative splicing of progerin pre-mRNA. Introduction of a short (25-mer) antisense morpholino oligonucleotide that can sterically block the cryptic splice site in exon 11 of progerin pre-mRNA resulted in a concentration-dependent decrease in progerin mRNA and protein levels and in the reversion of cellular phenotypes in cultured HGPS cells (Scaffidi and Misteli 2005). A similar strategy was successfully tested in vivo in *Zmpste24*^−/−^ and *Lmna*^*G609G/G609G*^ progeria mice, resulting in improved body weight, extended lifespan and improvement of several HGPS phenotypes (Osorio et al. 2011). This finding, together with the increasing evidence that the use of oligonucleotides for correction of splicing defects has growing therapeutic applications, initiated a set up of a new clinical trial that is currently under design (Cau et al. 2014).

Finally, a few approaches have been described to target cellular HGPS phenotypes. Resveratrol, an activator of SIRT1, a deacetylase involved in many cellular processes (Lavu et al. 2008), was found beneficial in the treatment of *Zmpste24*^−/−^ mice (Liu et al. 2012). Resveratrol treatment was previously shown to increase life span in yeast, worms, and flies and to enhance health span in rodents (Baur et al. 2006; Howitz et al. 2003; Milne et al. 2007; Wood et al. 2004). The mechanism of the beneficial effect of resveratol in HGPS mice is unclear, but it has been shown that SIRT1 interacts with lamin A in wild-type cells (Liu et al. 2012). In the presence of progerin or prelamin A, SIRT1 exhibits reduced association with the nuclear matrix and decreased deacetylase activity, leading to rapid depletion of adult stem cells in *Zmpste24*^−/−^ mice. Resveratrol enhances the binding of SIRT1 to A-type lamins, increases SIRT1 deacetylase activity and slows down body weight loss and significantly extends the life span of *Zmpste24*^−/−^ mice (Liu et al. 2012). However, another study conducted with an osteoblast and osteocyte-specific progerin-expressing mouse model (Schmidt et al. 2012) did not reveal a beneficial effect of resveratrol (Strandgren et al. 2015). Thus, more detailed studies are needed to find out whether resveratrol is a potentially promising drug for treatment of HGPS.

## Concluding remarks

The number of molecular biological studies aiming at the identification of lamin-mediated molecular disease mechanisms involved in HGPS increased tremendously following the surprising discovery that *LMNA* is causally linked to the premature aging disease HGPS in 2003. Despite numerous cellular pathways that were identified to be affected by the expression of the mutant lamin A protein (Fig. 2), the mechanistic details behind these effects are still unclear in most cases. Knowledge based on what was already known on lamin biology before the protein was linked to HGPS and findings on novel roles of lamins in diverse pathways in recent years allowed the launch of translational studies and the efficient search for drug targets and therapeutic approaches within a short time period. The results of the first clinical trials taught us that some improvements of the disease phenotypes can be achieved by FTI treatment, but they also made clear that we need a much better understanding of the underlying disease mechanisms to be able to tackle specific aspects of the disease in a more focused approach. It will also be important to elucidate which of the numerous pathways found to be impaired in HGPS are most relevant for and causally involved in the pathologies, and which ones are just bystanders.
